# Extraction of Pectin from Passion Fruit Peel: Composition, Structural Characterization and Emulsion Stability

**DOI:** 10.3390/foods11243995

**Published:** 2022-12-09

**Authors:** Yonglun Liang, Yang Yang, Lili Zheng, Xiaoyan Zheng, Dao Xiao, Shenwan Wang, Binling Ai, Zhanwu Sheng

**Affiliations:** 1 Haikou Experimental Station, Chinese Academy of Tropical Agricultural Sciences, Haikou 571101, China; 2 Haikou Key Laboratory of Banana Biology, Haikou 571101, China

**Keywords:** pectin, extraction, passion fruit, steam explosion, ultrasonic, emulsion stability

## Abstract

Extraction methods directly affect pectin extraction yield and physicochemical and structural characteristics. The effects of acid extraction (AE), ultrasonic-assisted acid extraction (UA), steam explosion pretreatment combined with acid extraction (SEA) and ultrasonic-assisted SEA (USEA) on the yield, structure, and properties of passion fruit pectin were studied. The pectin yield of UA was 6.5%, equivalent to that of AE at 60 min (5.3%), but the emulsion stability of UA pectin was poor. The pectin obtained by USEA improved emulsion stability. Compared with UA, it had higher protein content (0.62%), rhamnogalacturonan I (18.44%) and lower molecular weight (0.72 × 10^5^ Da). In addition, SEA and USEA had high pectin extraction yields (9.9% and 10.7%) and the pectin obtained from them had lower degrees of esterification (59.3% and 68.5%), but poor thermal stability. The results showed that ultrasonic-assisted steam explosion pretreatment combined with acid extraction is a high-efficiency and high-yield method. This method obtains pectin with good emulsifying stability from passion fruit peel.

## 1. Introduction

Passion fruit, in increasing demand yearly, is widely grown in many countries. The fleshy passion fruit pulp is commonly used to prepare juices and jams [1,2,3]. During this production, many by-products are produced, especially the peel, which accounts for about 50–60% of the total fruit mass. How to use and improve the value of passion fruit peel has attracted much attention [4].

Passion fruit peel is rich in pectin. Pectin is a complex polysaccharide, mainly composed of a galacturonic acid (GalA) main chain and a neutral sugar side chain. Its main domains are homogalacturonans (HG), rhamnogalacturonan I (RG–I) and rhamnogalacturonan II (RG–II) [5]. GalA residue in the HG backbone can be methylated at C–6 or acetylated at O–2 and/or O–3 [6]. Pectin is often used as a gelling agent, thickener and stabilizer in food, pharmaceuticals and cosmetics [7]. Passion fruit peel is a good source for extracting pectin. Scholars have obtained pectin with an extraction yield of 6.20–18.2% from passion fruit peel through different extraction methods [8,9,10,11,12].

Acid extraction (AE) is a traditional commercial pectin extraction method. Its process is mature, but the extraction time is longer than 60 min, and its performance cannot fully meet the needs of modern industry. Therefore, scholars have developed many novel pectin extraction technologies, such as ultrasound, microwave, ohmic heating, pulsed electric field, high pressure, high-speed shearing and other emerging technologies and their combinations [12,13]. Ultrasonic-assisted acid extraction (UA), a method to improve resource efficiency, has been applied to the extraction of pectin by scholars. De Oliveira et al. [10] obtained pectin with an extraction yield of 12.7% from passion fruit peel using the UA method. Tran et al. [14] obtained pectin with a yield of 53.8% from papaya at a UA time of 35 min, higher than AE for 100 min. Maran et al. [15] used a UA time of 27 min to obtain pectin with a yield of 9.0% from *Musa balbisiana*.

Steam explosion (SE) is an efficient new heat treatment technology [16]. The principle of SE is that biomass undergoes high-pressure steam and then instantaneously depressurizes, opening the biomass structure and releasing small molecular substances [17]. Studies have shown that SE has a high extraction yield in the extraction of bioactive components, such as phenols [18], flavonoids [19] and monosaccharides [20].

Pectin is gradually being accepted as a food emulsifier. The emulsifying and emulsion stability properties of pectin have attracted much attention in recent years. The emulsifying properties of pectin are closely related to the extraction method and the source of pectin [21]. Schmidt et al. [22] studied citrus pectin’s interfacial and emulsifying properties with different esterification degrees. Chen et al. [23] obtained pectin with excellent emulsification at a low concentration by the AE method from fruit dragon peel. Liu et al. [24] compared the structure and emulsifying properties of Gaertn seed pectin, apple pectin and citrus pectin. In summary, the emulsifying properties of pectin are related to the domain, protein content, degree of esterification, molecular weight and other pectin structures of pectin molecules [25].

This study compared the pectin extraction yield of AE, UA, steam explosion pretreatment combined with acid extraction (SEA) and ultrasonic-assisted SEA (USEA). Then, the following research was conducted: (1) characterization of the protein content, total phenol content (TPC), degree of esterification (DE), morphological characteristics, molecular weight (M_w_), Fourier transform infrared (FT-IR) spectroscopy and X-ray diffraction (XRD) of pectin; (2) determination of pectin’s thermal and emulsifying properties and clarification of the relationship between properties and structure. This work aims to obtain a pectin extraction method from passion fruit peel with high yield and good emulsifying stability.

## 2. Materials and Methods

### 2.1. Materials

Purple passion fruit peel was collected in Haikou, China. The passion fruit peel was cut into small pieces, treated in a water bath at 90 °C for 3 min and dried at 45 °C for 48 h. The dried peel was crushed and collected with a 40-mesh sieve to obtain the dried peel powder. GalA, arabinose (Ara), galactose (Gal), rhamnose (Rha), xylose (Xyl), glucose (Glc), mannose (Man) and other standards were purchased from Sigma-Aldrich (Shanghai) Trading Co., Ltd., Shanghai, China. Other chemicals and solvents are of analytical grade.

### 2.2. Pectin Extraction

#### 2.2.1. Acid Extraction (AE)

The AE process of pectin followed the method of Kulkarni et al. [26] with slight modifications. The dried peel powder was treated with a ratio of 1:30 HNO_3_ (pH 2.0) at 98 °C for 60 min. After vacuum filtration, the filtrate was concentrated by rotary evaporation to a quarter of the original volume. Then, a 3-fold volume of ethanol (95% *v*/*v*) was added and stood for 24 h to precipitate pectin. After centrifugation at 4000× *g* for 10 min, the residue was redissolved with an appropriate amount of distilled water and loaded in a dialysis bag (20,000 Da). The bag was placed into a large beaker containing a large amount of distilled water dialyzed for 48 h, concentration by rotary evaporation and freeze drying were performed to obtain purified pectin.

#### 2.2.2. Ultrasonic-Assisted Acid Extraction (UA)

UA of pectin was performed according to the method of de Oliveira et al. [10] and slightly modified. The dried peel powder was treated with a ratio of 1:30 HNO_3_ (pH 2.0) by an ultrasonic cleaner (JP-080B, Jiemeng Cleaning Equipment Co., Ltd., Shenzhen, China) under 480 W and 65 °C continuous ultrasound for 15 min. Pectin precipitation, purification and drying steps were the same as in Section 2.2.1.

#### 2.2.3. Steam Explosion Pretreatment Combined with Acid Extraction (SEA)

Pretreatment of dried peel refers to the method of Zhang et al. [27] with minor modifications. Dried peel (70 g) broken up in advance was loaded into the reaction chamber of an SE unit (QBS-80; Zheng Dao Co., Ltd., Hebei, China) and then the reaction door was closed to create a closed space. The peel was placed under steam at 0.6 MPa for 120 s, then the explosion was completed at 0.0875 s. After SE treatment, the sample was dried at 45 °C for 48 h and through a 40-mesh sieve after high-speed crushing. The pectin extraction steps were the same as in Section 2.2.1.

#### 2.2.4. Ultrasonic-Assisted Steam Explosion Pretreatment Combined with Acid Extraction (USEA)

SE pretreatment of peel reference Section 2.2.3 and pectin extraction steps were the same as in Section 2.2.2.

### 2.3. Yield

The yield of pectin was calculated with reference to Muñoz-Almagro et al. [28]. The yield of pectin was calculated using the following formula:

(1) $$\mathrm{Yield}\;\left(\%\right)=\frac{\mathrm{weight}\;\mathrm{of}\;\mathrm{dried}\;\mathrm{pectin}}{\mathrm{weight}\;\mathrm{of}\;\mathrm{dried}\;\mathrm{powder}}\times 100$$

### 2.4. Protein Content and TPC

Determination of protein content in pectin was based on protein binding to Coomassie brilliant blue G-250 with maximum light absorption at 595 nm [29]. TPC determination was the gallic acid equivalent obtained using the Folin–Ciocalteu colorimetric method [30].

### 2.5. Structure Analysis of Pectin

#### 2.5.1. Scanning Electron Microscopy (SEM)

The morphology of the pectin was observed by an SEM (S-3000N, Hitachi Co., Ltd., Tokyo, Japan). Pectin was fixed on the sample stage and sprayed with a thin layer of gold. Next, the pectin samples were placed under an SEM at an accelerating voltage of 10.0 kV.

#### 2.5.2. Determination of Degree of Esterification (DE) Value

The DE of pectin was determined following the method of Kazemi et al. [31]. Pectin (0.2 g) was wet with 2 mL of anhydrous ethanol and diluted with distilled water to 20 mL. Five drops of phenolphthalein indicator solution were added and titrated with 0.1 M NaOH until pink appeared and did not fade within 30 s as the titration endpoint. The volume of consumed NaOH solution was recorded and denoted as V_1_. Then, 10 mL of 0.1 M NaOH solution was added to a thermostatic oscillator, shaken at 180 rpm for 2 h at 30 °C and 10 mL of 0.1 M HCl solution was added as the end. After adding 5 drops of phenolphthalein indicator solution, the pectin was titrated with 0.1 M NaOH solution until pink appears and does not fade within 30 s. The consumption of NaOH was recorded, denoted as V_2_. The DE of pectin was calculated using the following equation:

(2) $$\mathrm{DE}\;\left(\%\right)=\frac{V_{2}}{V_{1}+{\;V}_{2}}\times 100$$

#### 2.5.3. Monosaccharides Composition

The monosaccharide composition of pectin was tested and slightly modified according to the method of Hosseini et al. [32]. Pectin was hydrolyzed with 2 M trifluoroacetic acid (TFA) solution at 100 °C for 2 h. Methanol was added and blow dried with nitrogen. The dried sample was redissolved with distilled water. We tested using high performance liquid chromatography (HPLC) equipment (Agilent 1260, Agilent Technologies Co. Ltd., Santa Clara, CA, USA) with an AGILENT EC-C18 column (2.1 mm × 50 mm, 2.7 μm) and RI detector. We used 0.005 M sulfuric acid as the mobile phase (the flow rate and temperature of the mobile phase were 0.8 mL/min and 70 °C, respectively). GalA, Ara, Gal, Rha, Glc, Gal, Xyl and Man were used as standard. The proportion of HG and RG–I regions of pectins were calculated according to Yang et al. [6], using the following formula:

(3) HG =GalA − Rha 

(4) $$\mathrm{RG}–I=\left(\mathrm{GalA}-\mathrm{HG}\right)+\mathrm{Rha}+\;\mathrm{Gal}\;+\;\mathrm{Ara}$$

#### 2.5.4. Molecular Weight (M_w_)

The M_w_ of the pectin was tested using an HPLC apparatus (Waters e2695, Waters Co., Ltd., Milford, MA, USA) equipped with two super hydrogel linear columns (7.8 mm × 300 mm) and connected in series with the Waters 2414 RI detector. The flow rate of the mobile phase (0.05% sodium azide solution) was 0.6 mL/min at 40 °C.

#### 2.5.5. FT-IR Spectroscopy

The sample was dried to constant weight, and about 1 mg of pectin was mixed with potassium bromide at a ratio of 1:50 and then tableted. The infrared region of 400–4000 cm^−1^ in the FT-IR spectrometer (Tensor27, BRUKER Co., Ltd., Karlsruhe, Germany) was scanned 16 times with a scanning resolution of 4 cm^−1^.

#### 2.5.6. XRD Measurement

The crystal structures of the samples were determined using XRD-6100 (SHIMADZU, Kyoto, Japan) at an accelerating voltage and current of 40 kV and 30 mA. The X-ray diffraction pattern is scanned at a diffraction angle (2θ) of 10–60° to obtain data at a scan rate of 4°/min.

### 2.6. Thermal Analysis

The thermodynamic characteristic (TG/DTG analysis) was measured at a range of 5–600 °C using a thermogravimetric analyzer (Q600, TA Instruments Co., Ltd., New Castle, DE, USA). The rate of temperature rise was 10 °C/min and at a nitrogen flow rate of 50 mL/min. The temperature and weight of sample changes were continuously recorded in the heating range.

### 2.7. Emulsifying Properties

The emulsion was prepared according to the method of Jiang et al. [33]. The completely dissolved pectin solution was set to gradient concentrations (0.5%, 1.0%, 1.5% and 2.0%, *w*/*v*). Ten milliliters of pectin solution was mixed with the same amount of corn germ oil, and then homogenized in a high-speed shearing homogenizer (A25, OuHor Mechanical Equipment Co., Ltd., Shanghai, China) at 14,000 rpm for 2 min to obtain the emulsion. A digital camera and microscopy observed the differences in emulsion stored in the dark at room temperature (23–27 °C) for 1 h and 10 days. An upright microscope (Axio scope A1, Carl Zeiss Co., Ltd., Oberkochen, Germany) was used to photograph emulsion droplets on the upper layer (2 cm below the liquid level) after 10 days of storage at room temperature at a magnification of 200×.

### 2.8. Statistical Analysis

Significant differences were obtained by analysis of variance (ANOVA) followed by Tukey’s test (*p* < 0.05). All tests were conducted in triplicate, and the results were expressed as mean ± standard deviation.

## 3. Results and Discussion

### 3.1. Effect of Extraction Method on Yield

The extraction yield of pectin is shown in Table 1. The extraction yield of pectin by different methods decreased in the order USEA (10.72%) > SEA (9.93%) > UA (6.52%) > AE (5.28%). Notably, the extraction yield is based on the calculation after dialysis purification. The extraction yield of pectin was greatly improved after SE pretreatment of passion fruit peel. The high-pressure steam treated by SE degraded some biomass components of the peel, reduced the internal bond or network strength of the peel molecules and completed the instantaneous thermal expansion impact material during the pressure relief stage, resulting in material breakage. The extraction yield of AE and UA has no statistical difference, but the extraction time was much lower for UA (15 min). The cavitation, thermal and mechanical effects of ultrasound destroyed the cell wall of the peel and improved the mass transfer efficiency [34,35]. Therefore, the USEA technology is an efficient and high extraction yield method.

### 3.2. Protein Content and TPC

As shown in Table 1, the protein content and TPC of pectin using USEA and SEA were significantly higher than those of UA and AE. At the same time, the ultrasonic process had only a small effect on protein content and TPC. Studies have shown that pectin protein positively impacts its emulsifying properties and TPC content closely affects the antioxidant properties of pectin [6,32]. Therefore, USEA technology may be one way to improve pectin properties.

### 3.3. Structural Characteristics of Pectin

#### 3.3.1. Morphology

**Figure 1:**
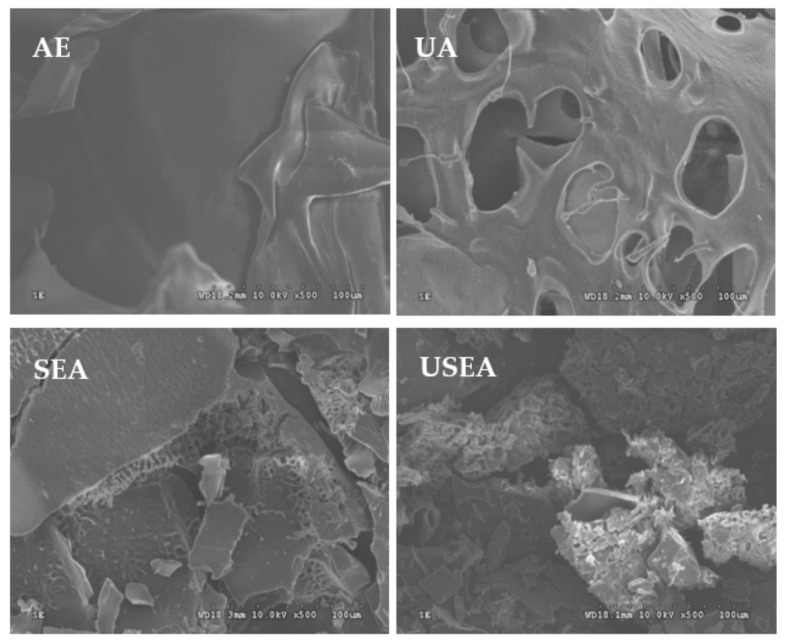
Morphology of pectin (×500).

The morphology of pectin is shown in Figure 1. The surfaces of pectin extracted by AE and UA are smooth, but there are some typical ultrasonic deep holes in the UA. The surface of by SEA was uneven and a certain degree of collapse occurred, accompanied by a certain degree of fragmentation. The pectin extracted by USEA underwent severe morphological changes, pits appeared to develop on the surface, as well as intense wrinkles, while producing many small fragments.

#### 3.3.2. DE

The DE value is related to the physicochemical properties of the extracted pectin. As shown in Table 2, the DE value of pectin recovered from passion fruit peel was 59.31–83.40%, which belongs to high methoxy pectin [31]. DE of pectin by UA was significantly higher than that of AE, similar to the results reported by Yang et al. [36], which may be due to the lower temperature and shorter extraction time of UA in this experiment [10,37]. The DE values of pectin extracted using SEA and USEA were significantly lower than those of AE and UA, respectively, which attributed to the high temperature and high-pressure environment during SE pretreatment [38]. In this study, SE pretreatment did not make passion fruit pectin become low methoxy pectin, but it significantly reduced the DE value of pectin. The technology of USEA may positively affect the de-esterification of pectin.

#### 3.3.3. Monosaccharide Composition and M_w_

Table 2 shows the monosaccharide composition and M_w_ of pectin. GalA accounts for 74.66–82.90% of passion fruit pectin monosaccharides. In passion fruit pectin, HG accounted for the main advantage, indicating that passion fruit pectin is rich in HG (linear) structure. The HG content of pectin by different extraction methods decreased in the order UA > USEA > AE > SEA. In contrast, the results of RG–I, opposite to HG, indicate that SE pretreatment reduced the linear structure of pectin and increased the hair area. Among them, the pectin extracted by UA and USEA have more linear structures than AE and SEA, respectively, because the ultrasonic process may lead to the degradation of pectin side chains [39]. The GalA/Rha ratio shows the difference in the main chains of pectin and the (Ara + Gal)/Rha reflects the average size of the neutral sugar side chains of pectin [40]. The (Ara + Gal)/Rha of pectin using USEA was the lowest, indicating that the side chain degradation of the combined process was the most serious. This difference in side chains may affect the thermal stability of pectin [30].

The functional properties of pectin are closely related to M_w_. The mass dispersion (M_w_/M_n_) of all pectin was low, indicating that the M_w_ of pectin was concentrated. The M_w_ of pectin by SEA and USEA were 0.74 × 10^5^ Da and 0.72 × 10^5^ Da, which were much smaller than AE (6.54 × 10^5^ Da) and UA (7.18 × 10^5^ Da). It seems that high molecular weight pectin was degraded to low molecular weight during SE pretreatment. The decrease in M_w_ of pectin may be related to the thermal degradation and the mechanical damage of instant decompression during SE pretreatment [18,41]. Combined analysis of monosaccharide composition M_w_ showed that the side chain and main chain of pectin extracted by SEA and USEA were seriously degraded at SE pretreatment.

#### 3.3.4. FT-IR and XRD Analysis

**Figure 2:**
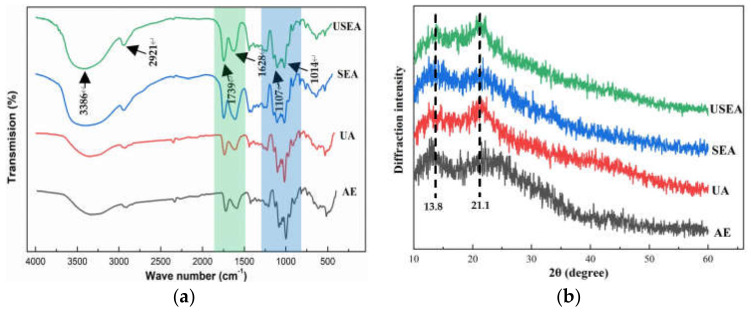
FT-IR spectra (**a**) and XRD patterns (**b**) of pectin.

FT-IR spectroscopy is a standard method to study the changes in pectin functional groups. The passion fruit pectin has a typical pectin FT-IR spectrum, as shown in Figure 2. The intense absorption peak near 3386 cm^−1^ and the weak absorption peak at 2921 cm^−1^ is generated by the stretching vibration of O–H and C–H (including CH, CH_2_, CH_3_) of pectin [42]. The absorption peaks near 1739 cm^−1^ and 1628 cm^−1^ were produced by the absorption esterification of esterified carboxyl (–COOR) and ionized carboxyl (–COO–), respectively. The “fingerprint region” of polysaccharides was 800–1300 cm^−1^, and the bands between 1107 cm^−1^ and 1014 cm^−1^ were related to furanose and α–and β–pyranose rings in pectin samples [43]. The fingerprint regions of pectin extracted by SEA and USEA were similar, with more pyranose rings than AE and UA.

Figure 2 shows the XRD pattern of passion fruit pectin. The passion fruit pectin has a semi-crystalline structure. There are two broad peaks near 13.8° and 21.1°. In addition, a small part of the crystal structure is shown at 21.7°, 22.9° and 12.5°. The results are similar to those reported for pectin from sunflowers [44] and sweet lemon [45]. The crystal structures of pectin extracted by AE and SEA are similar but different from UA and USEA. It shows that SE pretreatment has little effect on the crystal structure of pectin, while the ultrasonic-assisted process changes the crystal structure of pectin. The difference in peak shape of XRD patterns between ultrasonically and thermally treated pectin was also reported [46].

### 3.4. Thermal Analysis of Pectin

**Figure 3:**
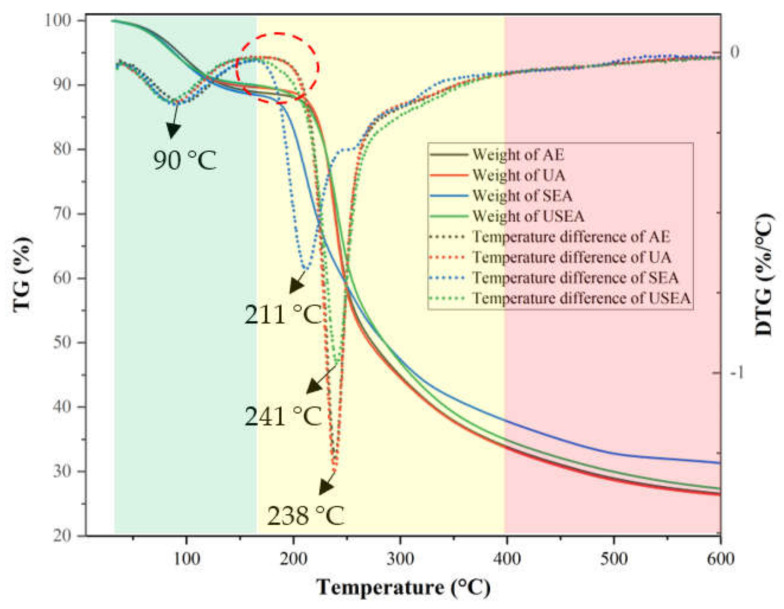
Thermodynamic characteristic of pectin.

The thermal analysis provides information on the thermal stability of polymer materials [47]. Figure 3 shows the TG and DTG curves of different passion fruit pectin. The thermal weight loss of pectin was divided into three regions: 50–160 °C, 160–400 °C and 400–600 °C. In the first region, the weight of pectin is slightly reduced and the maximum mass loss speed was at nearly 90.0 °C, shown in the DTG image. It is caused by the evaporation of free water and bound water in pectin [48]. In the second region, the weight of pectin extracted by AE, UA and USEA decreased by 55.2%, 56.1% and 55.2%, respectively, while SEA decreased by 50.8%. For DTG, the maximum mass loss temperatures of pectin obtained from AE and UA were 238 °C, while those of SEA and USEA were 211 °C and 241 °C. It should be noted that although the maximum mass loss speed temperatures of SEA and USEA were lower than those of AE and UA, their loss began at a lower temperature. The pectin obtained by USEA and SEA was more susceptible to thermal degradation mainly due to the decrease of M_w_ of pectin by SE treatment [23]. The change in the second region can be explained by the thermal degradation of the galacturonic acid chain, the decarboxylation of the acid side group and the carbon in the ring to generate different gaseous products and solid char [49]. The third region shows a slow weight loss, which may be due to the slow thermal decomposition of solid carbon due to the continuous temperature increase. In general, SE pretreatment reduced the thermal stability of passion fruit pectin.

### 3.5. Emulsifying Properties of Pectin

**Figure 4:**
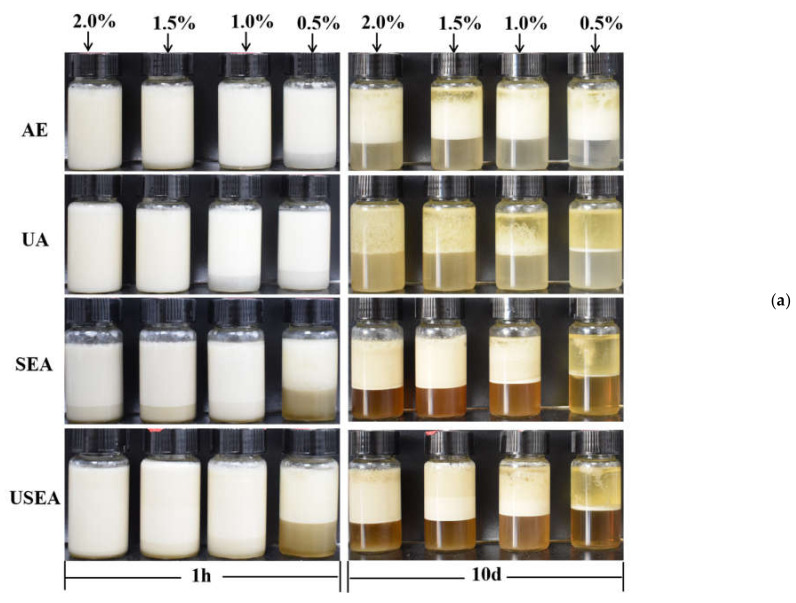

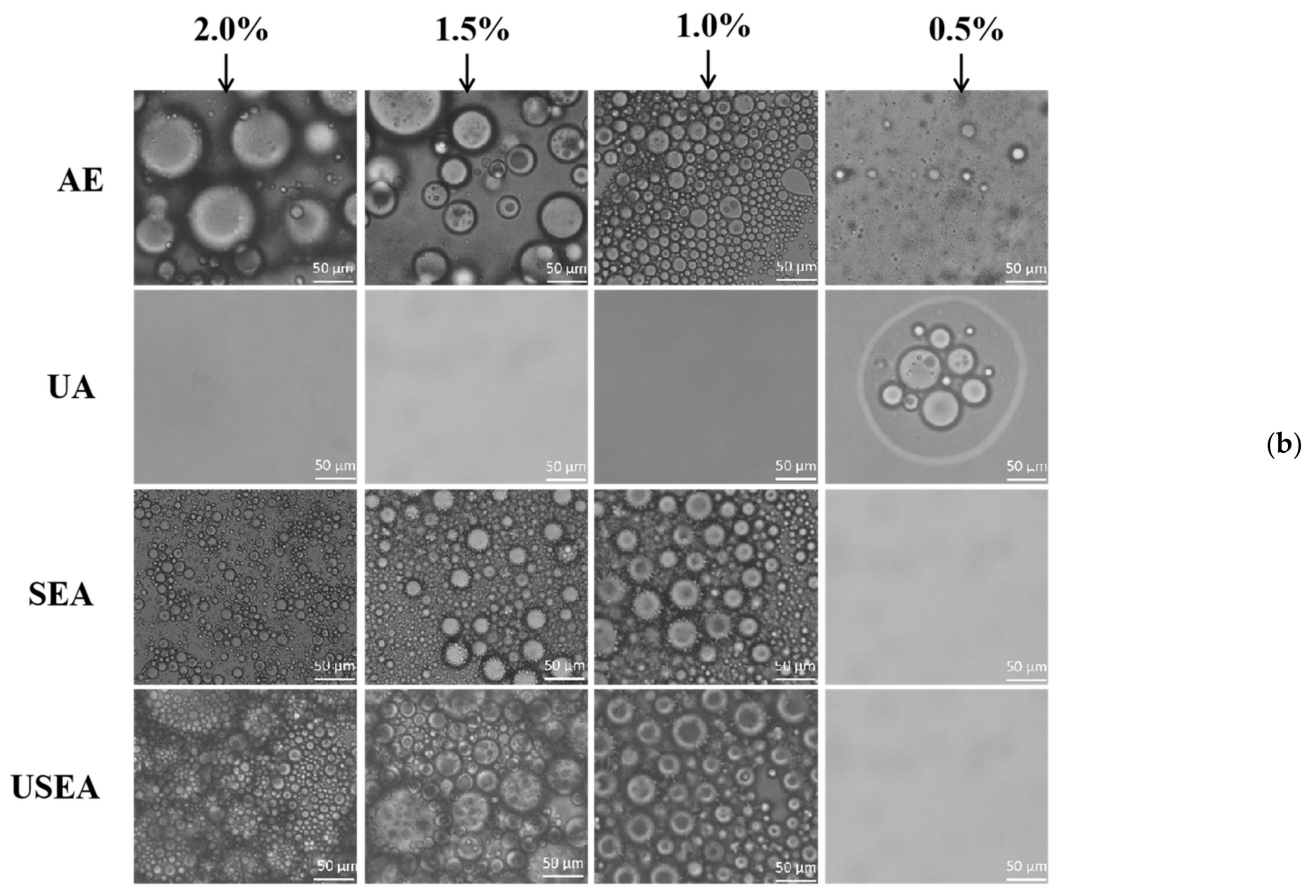
Emulsifying properties of pectin. (**a**) The appearance of emulsions after storage for 1 h and 10 days at room temperature; (**b**) optical microscope images of droplets in the upper layer of the emulsions after storage for 10 days at room temperature (×200).

The digital photos of the emulsions after storage for 1 h and 10 days at room temperature are shown in Figure 4a. After 1 h of preparation, some pectin emulsions (AE–0.5%, UA–1.0%, UA–0.5% and all SEA and USEA pectin emulsions) had different degrees of phase separation. During the storage process (Figure S1), it can be seen that the creaming index of pectin emulsions changed significantly in the short term after preparation and related to the concentration of pectin. It is worth noting that the pectin emulsions (except for UA) had little change in the creaming index during subsequent storage. The higher concentration of pectin led to a higher stability of emulsions only in short timeframes due to the increase in viscosity. However, the concentration did not have an effect at longer times [33]. For SEA and USEA pectin emulsions, more phase separation occurred in the early stage of storage, which can be resolved as the lower viscosity of the pectin emulsion was caused by lower M_w_. In addition, the pectin emulsion stability of UA was lower than that of other methods, which may be related to its lower RG–I content [23].

Figure 4b shows optical microscope images of droplets in the upper layer of the emulsions after storage for 10 days at room temperature. The pectin emulsions of UA can only be observed with less or no droplets. However, the pectin emulsions of SEA and USEA can still see dense and tiny droplets in the concentration of pectin solutions at 1.0%, 1.5% and 2.0%. According to Stoke’s law, the pectin emulsions of SEA and USEA with smaller droplet sizes may have better emulsion stability [33]. The emulsion stability of SEA and USEA pectin during long-term storage may be related to their high protein and RG–I content which mediates the intermolecular association between adsorbed pectin chains to prevent droplet coalescence [50].

## 4. Conclusions

This study compared four processes for extracting pectin from passion fruit peel. The UA process improved the extraction efficiency and obtained a higher DE value and M_w_ pectin but reduced the proportion of RG–I of pectin, resulting in the pectin emulsion stability not being as good as that of the AE. SE pretreatment significantly improved the extraction yield of pectin and obtained a lower DE value and lower M_w_ of pectin (SEA and USEA). The pectin extracted by SEA and USEA has higher protein content, TPC and RG–I structure and has better emulsion stability, but reduces the thermal stability of pectin. In general, USEA combines the high efficiency of UA and the high extraction yield of SEA, and the pectin obtained by this method has good pectin emulsion stability. This study provides a pectin extraction method with a high yield and good emulsifying properties from passion fruit peel.

## Figures and Tables

**Table 1:** Extraction yield, protein content and polyphenol content of pectin.

Samples	Yield (%)	Protein Content (%)	TPC (mg GAE/g)
AE	5.28 ± 1.57 ^b^	0.46 ± 0.08 ^d^	8.58 ± 0.47 ^b^
UA	6.52 ± 0.48 ^b^	0.51 ± 0.05 ^c^	9.15 ± 0.35 ^b^
SEA	9.93 ± 0.33 ^a^	0.68 ± 0.04 ^a^	17.55 ± 0.55 ^a^
USEA	10.72 ± 0.39 ^a^	0.62 ± 0.01 ^b^	18.85 ± 0.22 ^a^

TPC, total polyphenol content; AE, acid extraction; UA, ultrasonic-assisted acid extraction; SEA, steam explosion pretreatment combined with acid extraction; USEA, ultrasonic-assisted steam explosion pretreatment combined with acid extraction. The meanings of AE, UA, SEA and USEA in subsequent charts and tables are described above. Values with different superscripts within a column are significantly different (*p* < 0.05).

**Table 2:** DE value, molecular weight and monosaccharide distribution of pectin.

Samples	AE	UA	SEA	USEA
DE (%)	74.50 ± 0.86 ^b^	83.40 ± 0.49 ^a^	59.31 ± 1.72 ^d^	68.49 ± 0.17 ^c^
M_w_ (×10^5^ Da)	6.54	7.18	0.74	0.72
M_w_/M_n_	1.80	1.65	1.65	1.67
Relative monosaccharide content (%, *w*/*w*)				
GalA	78.41	82.90	74.66	81.26
Ara	15.74	12.20	14.35	12.04
Gal	3.10	2.76	5.61	3.80
Rha	1.39	0.98	1.43	1.30
Xyl	0	0	0	0
Glc	0.82	0.68	2.01	1.04
Man	0.54	0.48	1.94	0.56
HG	77.02	81.92	73.22	79.96
RG–I	21.62	16.92	22.83	18.44
GalA/Rha	56.43	84.62	52.07	62.61
(Ara + Gal)/Rha	13.56	15.27	13.92	12.20

DE, degree of esterification; M_w_, molecular weight; M_n,_ number-average of molar mass; GalA, galacturonic acid; Ara, arabinose; Gal, galactose; Rha, rhamnose; Xyl, xylose; Glc, glucose; Man, mannose; HG, homogalacturonans; RG–I, rhamnogalacturonan I. HG = GalA − Rha; RG–I = GalA − HG + Rha + Gal + Ara. Values with different superscripts in the same row are significantly different (*p* < 0.05).

## Data Availability

Data is contained within the article or Supplementary Materials.

## Supplementary Materials

The following supporting information can be downloaded at: https://www.mdpi.com/article/10.3390/foods11243995/s1, Figure S1: The creaming index of pectin emulsions for 10 days at room temperature.
