# Supplementary material for: Extraction of Pectin from Passion Fruit Peel: Composition, Structural Characterization and Emulsion Stability
Source: Foods. 2022 Dec 9;11(24):3995. doi: 10.3390/foods11243995 (PMC9777908; doi:10.3390/foods11243995)
Supplement: Supplementary file 1 [file foods-11-03995-s001.zip › foods-2031188-supplementary.pdf]

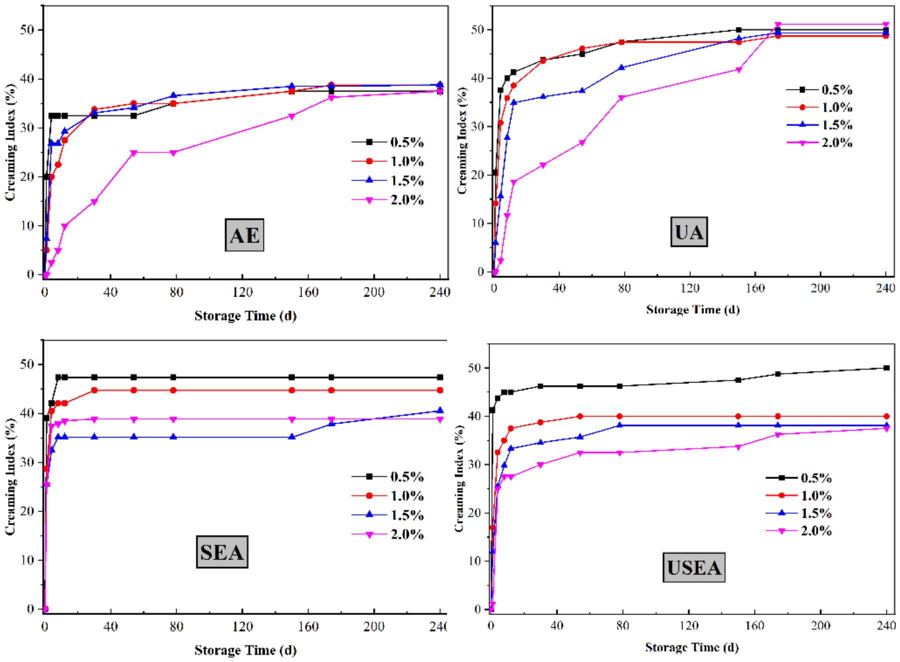

Figure S1 The creaming index of pectin emulsions for 10 days at room temperature.

The calculation of creaming index using the following formula:

$$\text{Creaming index (\%)} = \frac{H_s}{H_e}$$

where H_s is the height of the serum layer below the cream layer and H_e is the total height of the emulsion.
